# Benefits of Eccentric Training with Emphasis on Demands of Daily Living Activities and Feasibility in Older Adults: A Literature Review

**DOI:** 10.3390/ijerph20043172

**Published:** 2023-02-11

**Authors:** Ján Cvečka, Matej Vajda, Alexandra Novotná, Stefan Löfler, Dušan Hamar, Matúš Krčmár

**Affiliations:** 1 Hamar Institute for Human Performance, Faculty of Physical Education and Sports, Comenius University in Bratislava, 814 69 Bratislava, Slovakia; 2 Ludwig Boltzmann Institute for Rehabilitation Research, 1100 Vienna, Austria; 3 Department of Physical Education and Sports, Faculty of Education, Constantine the Philosopher University in Nitra, 949 74 Nitra, Slovakia

**Keywords:** eccentric exercise, physical demands, elderly, daily living tasks, injury prevalence

## Abstract

Aging is associated with a decline in physical capabilities and several other health-related conditions. One of the most common age-related processes is sarcopenia. Sarcopenia is usually accompanied with a decline in skeletal muscle mass and physical functioning. A decrease in these markers usually impacts basic daily living activities (DLAs), which become somewhat harder to accomplish for older individuals. Several research studies have examined the demands of DLA in older individuals with results indicating that activities such as walking, sitting, standing, stair climbing, stair descending, and running generate high demands on older adults. The forces that act on individuals are in most cases equal or multiple times higher relative to their body mass. For instance, it was reported that the GRF (ground reaction force) during stair descent ranged from 1.43 to 1.50 of BW (body weight) in an older population. Even higher demands were recorded during other related activities. These demands of DLA raise the question of appropriate rehabilitative or training management procedures. During the past decades, an eccentric form of resistance training gained popularity due to its effectiveness and lower metabolic demands, which seems to be an appropriate method to develop and maintain a basic level of strength capabilities in higher age. Multiple factors of eccentric training have been examined including modality of exercise, intensity, frequency, and safety of the elderly. Several modalities of eccentric exercise have been shown to be effective including traditional methods, as well as machine-based ones, with or without using some equipment. The studies included in this review varied in intensity from low to high; however, the most frequently used intensity was ≥50% of the maximal eccentric strength during two or three eccentric sessions per week. Importantly, the prevalence of injury of older adults appears to have been low to none, highlighting the safety of this approach. In summary, eccentric training prescriptions for older adults should consider the demands of DLA and the characteristics of the elderly for appropriate management of training recommendations.

## 1. Introduction

An aged population is already a current problem and no longer a future perspective. Demographic aging has risen rapidly since the middle of the 20th century, especially in economically developed countries. This trend or current situation is mostly caused by improved healthcare, hygiene, and nutrition, as well as higher living standards. In 2015, the number of people over the age of 60 was 901 million (12% of the world’s population). Forecasts of the future demographic development of the world’s population over the age of 60 speak to a 3.6% increase per year, which will represent 1.4 billion in 2030 and 2.1 billion in 2050 [1]. This forecast was confirmed by a WHO study [2], according to which the population over the age of 60 will almost double to 22% of the world’s population.

As we age, there is a natural age-related process known as sarcopenia. Sarcopenia is frequently defined as a loss of skeletal muscle mass and related functions [3]. According to the work of the European Working Group on Sarcopenia in Older People (EWGSOP), sarcopenia comprises three components: low level of muscle mass (≤8.90 kg/m^2^ in men and ≤6.37 kg/m^2^ in women), decreased muscular strength usually assessed by handgrip strength (<30 kg in men and <20 kg in women), and slowed walking speed (≤0.8 m/s) [4].

Briefly, among the potential mechanisms causing this process, the following can be considered: loss of motor units [5], drop in hormone production (e.g., testosterone and growth hormone) [6], expansion of subcutaneous and intermuscular adipose tissue [7], and chronic inflammation, which may contribute to skeletal muscle loss and physical functions [8]. In a recent meta-analysis by Steffi et al. [9], the authors found a significant association between level of physical activity and sarcopenia development, and these results were consistent in most of the examined studies. Irrespective of still fully unknown mechanisms that potentially underlie the process of sarcopenia, it seems that the general consensus on how to counteract this process lies in the incorporation of appropriate physical activity into the life of elderly people. This suggestion has already been confirmed by several research works that addressed multiple benefits of physical activity on the physical and health-related status of elderly people, including an increase in muscle mass/muscle fiber proportion [10,11]. The previous suggestion was also confirmed by another recent meta-analysis by Grgic et al. [12] who found that even very elderly individuals (>75 years) can significantly increase muscular strength, as well as muscle size.

The abovementioned predictions have also been confirmed by the increasing number of universities with programs focused on studying aging from many different perspectives: biological, social, mental, disease, and disability [13]. As a consequence of this situation, many research papers have begun to address the impact of various physical activities (especially resistance/strength training) to improve the quality of life and physical fitness of the elderly [14,15,16,17]. Complex positional statements have also been published on the effectiveness and integration of resistance training into the lives of the elderly population [18].

Another and more detailed aspect of resistance or strength training itself is a contraction type or mode of exercising. Regardless of the numerous studies focused generally on the effectiveness of traditional resistance exercise, there are also numerous studies that examined eccentric strengthening exercises themselves [19,20,21]. Apart from the mentioned ones, there are a considerable number of studies dealing with eccentric training, not only in healthy older population but also in people suffering from, for instance, locomotor restrictions, pulmonary diseases, and degenerative changes, or with other limitations or complications.

On the basis of the abovementioned studies, the research question is why assessing external demands during daily living activities or why this type of research can be helpful. As mentioned above, sarcopenia can impede daily living tasks, which become more demanding for older individuals. This suggestion was also confirmed by Perez-Souza et al. [22] who found a negative impact of sarcopenia on daily living tasks such as gait speed. On this basis, we can assume that these demands (especially the forces acting on the joints) are even higher during more difficult tasks that include the eccentric phase of movements, and knowing these demands can help us better manage eccentric training recommendations.

### 1.1. External Loads during Daily Living Activities: How Much Strength Do Older Adults Need?

This section is dedicated to a logical analysis of studies whose purpose was to find out external loads or forces which act on older individuals during daily living tasks such as walking, stair climbing, chair raise, and other similar activities which the elderly population performs on a daily basis. However, this section also provides a comparison to the younger population (as also presented in Table 1), as well as the usage of different metrics or devices across multiple studies. Table 1 provides an overall summary of studies included in this literature review.

Research in this area goes back several decades (e.g., [23,24,25,41]). For instance, Rauben and Siu [41] and Kowalk et al. [24] claimed that stair climbing is a typical daily living activity that differs from walking in terms of range of motion, muscle activity, and joint forces and moments produced. Rauben and Siu [41] reported that stair climbing was the hardest DLA according to multiple physical performance assessments in older individuals. Knowing how older individuals perform this activity and how lower extremities work can help with pathogenesis or lower-extremity disorders.

One of the reasons for determining the demands of this activity or similar ones is to prepare patients and suggest criteria for safe joint replacements of the lower-extremity, as well as rehabilitation [23].

Regarding forces and moments produced during stair climbing (ascending and descending), the comparison of achieved values across the studies is a difficult task due to the use of several different approaches and normalizations. For instance, Leitner et al. [25] performed three stair ascent and descent tasks in 44 female and 15 male old individuals at their self-selected normal speed, and ground reaction forces were measured by multicomponent force platforms. Vertical GRFs (VGFRs) for the right and left legs during stair ascent were 743.1 N and 735.9 N (second highest peak forces), respectively. During stair descent, VGFRs for the right and left leg were 1011.8 N and 953.9 N (highest peak forces), respectively. Vertical GRFs during stair descent exceeded the body weight of older subjects and could be considered a more challenging task compared to the stair ascent. These values were also considerably higher compared to standard walking activities. A comparison across studies is also challenging due to data in some studies being available only in the form of graphs [23]. Kowalk et al. [24], for instance, normalized values during stair ascent and descent to body weight and leg length and expressed them as a percentage However, the trend regarding demands during stair ascent and descent was comparable to Leitner et al.’s [25] study. The highest knee extension moments during stair ascent from the first to the second step ranged from 9.7 to 9.4%; when descending, the values ranged from 14.0 to 15.4%. It should be noted that, in the study by Kowalk et al. [24], young to middle-aged individuals were assessed; regardless, the demands (trends) of similar tasks were comparable to the study by Leitner et al. [25].

Christina-Cavan [26] examined stair descent in young and older populations. They normalized ground reaction forces (GRFs) to body weight. They found that GRFs during stair descent ranged from 1.43 to 1.50 BW (two highest force peaks) in the older population and from 1.40 to 1.48 BW (two highest force peaks) in the young population. It could be seen that overall load highly exceeded body weight in both populations, with a slightly higher load in older ones. The authors suggested that older individuals use different strategic modifications to minimize the dependence on frictional forces, as well as the probability of a lack of control during the descending phase (lowering the body). Similar demands during stair descent as in the study of Christina-Cavan [26] were found by Luepongsak et al. [33], who, in addition to stair descent, examined walking, standing, and chair raise in the elderly. They also found that, during stair descent, the GRFs were 123.58% of BW at the knee and 108.74% of BW at the hip, which were considerably higher values compared to the walking activity, where they found 101.03% of BW at the knee and 88.75% of BW at the hip joint. Similar results to the abovementioned studies were found by Stacoff et al. [34], where GRFs during stair descent ranged from 1.62 to 1.67 BW in young individuals and from 1.51 to 1.53 BW in older individuals. During level gait, values ranged from 1.22 to 1.23 BW in young and from 1.12 to 1.13 BW in older individuals. It should be noted that, in the study by Stacoff et al. [34], they also examined different slopes of stairs; hence, GRF outcomes normalized to BW were varied more depending on the slope. Data from these studies showed similar trends and demands regarding the mentioned activities; in the study by Taylor et al. [35], the authors also examined gait and stair climbing, but their methodology was based on a case study of selected subjects with total hip arthroplasty, and contact forces were measured not only by a standard force platform and video system but also in vivo (hip contact forces in the femoral coordinate system). The outcomes of tibiofemoral forces were much higher compared to previous studies, whereas, during walking, values ranged (four subjects) from 2.97 to 3.33 BW; during stair climbing, values ranged from 5.09 to 5.88 BW. The presented data clearly show that demands put on joints during daily living activities highly exceed patient or person body weight and, thus, can be considered as demanding, especially in older populations where the demands from these activities can be closer to their maximal produced force. This suggestion was also confirmed in the study by Hortobágyi et al. [36], who found that during tasks such as stair ascent and descent and chair raise, the relative effort during ascent was 58% and 78% in young and old adults, respectively. While descending, it was 42% and 88%, and, during the chair raise, it was 42% and 80% in young and old adults, respectively. Additionally, young adults achieved higher total strength, whereas older ones were much closer to their maximal capabilities in all activities. Similar findings were also found by Reeves et al. [42]. The authors suggested that older people are able to redistribute the joint moments in order to safely perform these activities.

Table 1 shows several research studies regarding the demands of daily living activities arranged chronologically. For instance, Kutzner et al. [37] examined the demands during various more common daily living tasks such as two-legged stance, sitting down, standing up, one-legged stance, level walking, stair ascent and descent, and knee bending. Their comprehensive analysis showed that the most demanding task was stair descent, where the measured force reached 400% of BW, followed by stair ascent (316% of BW), standing up, level walking, one-legged stance, and knee bend values, which ranged from 246% to 261% of BW, and finally two-legged stance with 107% of BW. This comprehensive analysis only confirmed previous studies on the demands on specific location/joint structures.

In a more recent study performed by Kulmala et al. [29], they examined and compared young and older populations during activities similar to those mentioned above, but they also included running, sprinting, and hopping tests to evaluate the joint demands of nonstandard tasks, which is interesting for older and more active populations. They measured peak ground reaction forces which were normalized to BW. The lowest demands were noted in the walking test (older: 1.27 BW, younger: 1.23 BW), followed by running (older: 2.85 BW, younger: 3.11 BW), sprinting (older: 2.93, younger: 3.76 BW), and hopping test (older: 2.93, younger: 3.76 BW).

In summary, the purpose of this section was to highlight specific and natural daily living activities that are an inseparable part of life of older people. The main goal of this section was not to deeply analyze the biomechanical or physiological understanding of differences and methodologies, but to outline these tasks in a broader manner to show that even so-called simple tasks in the everyday life of older people are very difficult and even more demanding. As shown in Table 1 and in the text, it is important to know how demanding these tasks are to be better prepared or to manage rehabilitation programs, especially today where very special attention is paid during training prescription to various eccentric strength training modalities as one of many tools that can improve and preserve health and overall function in older adults. In addition, the abovementioned demands of everyday activities are associated with some level of eccentric strength [43].

### 1.2. Eccentric Training: Benefits and/or of Possible Risks in Older Adults

In recent years, resistance or strength training, especially eccentric strength training, has become one of the most effective methods to counteract age-related changes of muscle mass and other structures [44]. Numerous studies have discussed the several benefits and unique physiological advantages of eccentric training compared to standard resistance training. There are probable suggestions that eccentric training is metabolically efficient (lower physiological demand) at a given workload with high work output [19,44,45]. Furthermore, some studies reported lower or minimal cardiorespiratory load evoked by eccentric exercise, which could be an advantage when applied for older individuals. Another aspect that needs to be considered before the application of eccentric exercise is delayed onset of muscle soreness (DOMS), which is usually manifested by pain, swelling, stiffness, and structural damage caused by eccentric contractions [46]. However, some authors have suggested that this phenomenon occurs less in older individuals. Lavender and Nosaka [47] examined the range of muscle damage caused by eccentric exercise in young and old men. They reported larger decreases in maximal voluntary contraction (MVC) and range of motion (ROM), along with increases in circumference, DOMS, and creatin kinase (CK), in young men compared to older ones. In addition, some authors have also suggested that this phenomenon occurs less in older individuals due to muscle fiber characteristics, in which type 2 fibers are reduced [47]. On the basis of the abovementioned studies, it seems that training strategies including eccentric forms of exercise reflect the physiological characteristics of older individuals and can be considered safe and effective to further facilitate positive changes in the older population. Through a literature review, we performed a database search of relevant studies where various forms of eccentric exercises/modes were analyzed, as further described in the next section. As presented above, eccentric exercise may be an alternative for older individuals, but it is important to consider multiple factors, such as modality, intensity, frequency/volume, and safety. So far, a considerable number of studies have focused on various modalities of eccentric exercise, as well as its intensity, frequency, and safety. These recommendations are based on the findings from research and, thus, may serve as a valuable resource for researchers and practitioners.

### 1.3. Eccentric-Based Training Modalities

There is currently an increasing number of studies focused on eccentric training in various areas (population groups). The usage of machines or modes for performing eccentric training varies across studies, predominantly as a function of rehabilitation practices, testing and training environments, and the characteristics of population involved. These machines range from well-known isokinetic devices based on motorized, pneumatic, and flywheel devices to those based on daily tasks such as stair descent or sitting down. There is also evidence of more frequent eccentric training using these machines in the older population and its effect on physical functioning. The rationale behind their usability lies in the fact that, in the older population, practitioners or researchers can better control intensity, range of motion, and safety during exercise. Several studies including older individuals used some of these machines and/or modes in training environments and evaluated their effect on physical functioning. For instance, Mueller et al. [20] applied eccentric training using custom-built eccentric bikes in older men and women. The results showed that subjects improved in the timed up and go test, as well as other outcomes such as body composition, muscle fiber type ratio, and eccentric coordination. The authors suggested that using these types of machines has favorable effect, and that they are well suited for the older population. Some studies investigated the effect of eccentric training using flywheel devices, which use inertia to overload the eccentric portion of the movement [44] in the older population [48,49,50,51]. Sañudo et al. [51] found significant improvements after flywheel training in physical functioning such as chair raise and walking speed. Similarly, Fernandez-Gonzalo et al. [49] applied flywheel eccentric training in older patients after stroke and found significant improvements in muscular power, balance, gait, and cognitive functions. Regarding isokinetic devices, La Stayo et al. [52] and Johnson et al. [53] applied the multi-joint Eccentron machine, which uses a motor to drive pedals in an alternating unilateral manner. In the study by Johnson et al. [53], the authors recorded significant improvements in functional tests such as chair raise, balance (one-legged stance), gait performance, and timed up and go test. La Stayo et al. [52] reported improvements in the 6 min walking distance and balance confidence; however, these changes were not statistically significant. Some studies focusing on the older population performed eccentric exercise using a mode that mimics daily living tasks without any equipment. For instance, Katsura et al. [54] examined muscular strength and functional fitness by applying manual resistance during the eccentric phase of movement in older adults. They found significant improvements in lower-body strength, 30 s chair raise, 3 min timed up and go test, and 2 min step test, as well as mobility improvements (sit and reach). Studies by Theodorou et al. [55] and Regnersgaard et al. [56] investigated the effects of stair descent and ascent training programs on force production, muscle mass, and functional measures such as the 6 min walk test. Both studies found significant improvements after stair descent training. These results indicate that even a simple activity such as stair descent, which is considered an eccentrically biased exercise, can serve as a prevention and treatment strategy to improve functional fitness and counteract sarcopenia in old adults. On the basis of the abovementioned studies, it can be concluded that various eccentric training modalities with or without equipment or performed on specific devices can be viable options for older individuals to improve functional/DLA outcomes.

### 1.4. Exercise Intensity

The intensity of eccentric exercise in most studies ranged from medium- to high-intensity training, whereas the intensity was typically ≥50% of the maximal eccentric strength, derived from the multiple repetition maximum of eccentric exercise or related to maximal concentric strength (>60% of concentric 1RM—one-repetition maximum) [19,21,57,58,59,60,61,62,63,64,65]. For instance, Johnson et al. [19] found significant improvements after eccentric training program with intensity ranging from 30% to 50% of the maximal eccentric strength in functional tasks such as 30 s chair raise, timed up and go test, and maximal eccentric strength in the community of dwelling older adults. The authors suggested that even a lower percentage of maximal eccentric strength can elicit positive adaptation related to daily living tasks of older individuals. In another study by Vincent et al. [21], eccentrically (load at 100% of concentric 1RM) focused resistance exercises were performed by older subjects on knee osteoarthritis and muscular strength. The results show significantly increased leg strength, and 50% of patients reported a significant reduction in pain. Raj et al. [59] divided participants into the conventional strength training and eccentric groups. The eccentric group trained with an intensity at 50% of 1RM unilaterally (both groups matched for total work). The results showed that the eccentric group improved in terms of muscular strength and the 6 min fast walking test. However, only the eccentric group further improved isometric torque and stair climbing, as well as the conventional group stair descent and timed up and go test, with a better performance in vertical jumping. On the basis of these results, it seems that the conventional method was superior compared to the eccentric one, but a couple limitations (as also authors pointed) should be noted: (1) the specificity of some tests such as vertical jumping, which comprises the concentric phase of movement; (2) potential underestimation of unilateral 1RM in the eccentric group. Interestingly, the eccentric group significantly increased quadriceps thickness and torque production at higher isokinetic velocities, which could have a positive impact on the older population prone to sarcopenia and falls. The intensity (in terms of load) of eccentric exercise seems to be an important factor not only when comparing different methods but also from a performance enhancement view. In a study by Dias et al. [62], subjects performed eccentric strength training with the total load at 75% of 1RM, whereas the eccentric group only slowed down the eccentric phase compared to the conventional group (4.5 and 1.5 s, respectively). Slowing down the eccentric phase led to significant improvements (no significant differences between groups) in knee extension 1RM, as well as the 6 min walk, stair climbing, and chair raise tests. In contrast, in the study by Katsura et al. [54], the authors applied eccentric training exercises without equipment or external load, focusing on the eccentric phase of the movement. The results showed significantly greater increases in muscle thickness, MVC, 30 s chair raise, timed up and go test, 2 min step test, and balance in favor of the eccentric group. This may suggest that even a lower intensity of eccentric training can enhance performance in daily living tasks. Similarly, significant improvements in the timed up and go test, lower leg strength, and muscle thickness were recorded in the study by Kay et al. [64], with a low to moderate RPE scale, after eccentric training with a load up to 50% of the MVC.

The intensity of eccentric training varied across the abovementioned studies. The intensity of eccentric exercise can elicit positive adaptations in outcomes that are relevant to everyday tasks and demands performed by older adults. This is also relevant to previous findings, where it was shown that eccentric exercising even with a higher intensity has a lower cardiorespiratory load, as well as a low to medium internal perception of the load/effort (measured by the RPE scale) required to complete these demands [64]. Overall, this can be beneficial for older individuals, especially when considering the health-related status of older adults and the rising number studies examining eccentric training and intensity in special or clinical settings, as well as its relevance to demands of daily living tasks, which, as presented above, can reach almost the maximum of the capabilities of some older individuals.

### 1.5. Frequency of Eccentric Trainings

Another factor to be considered when eccentric exercising is applied is the frequency of training sessions. The majority of studies applied frequencies ranging from two [19,21,59,62,63,64] to three [58,65] training sessions per week, with positive improvements in performance. We can also find several other studies with two [50,53,66] or three [52,67] eccentric training sessions, which came to similar conclusions regarding the positive changes in outcomes related to daily living activities such as walking, stair climbing, chair raise, and muscular strength. These research studies are consistent regarding eccentric training frequency and its benefits or improvements. Confirmation of this finding is very difficult as, to our knowledge, there are currently no meta-analytical studies focusing on eccentric training frequency and its overall effects. This warrants further research in this area in the older population. However, there are some indications (biased toward younger subjects) based on Crane et al.’s [68] study, who investigated high and low eccentric training frequency and found no significant differences between 1 vs. 3 days of eccentric sessions per week with matched volume. Possibly better insight into this area was provided in a systematic review by Borde et al. [69] with a focus on conventional strength training in the older population. The results suggested that training frequency up to three sessions per week seems to be optimal, with the best results. Further research, especially regarding eccentric training frequency, should be conducted in the older population to verify whether similar results are achieved to those obtained in conventional exercise or the younger population. However, the results from the studies provided suggest that eccentric training performed two times per week is best.

### 1.6. Safety and Injury Limitations

Many research studies with a varied number of participants of diverse backgrounds (healthy older individuals and older individuals with health problems or other limitations) have demonstrated the successful and safe performance eccentric training with continuously increasing intensity and volume over time [19,20,21,52,58,61,63,64,65,66,70,71,72,73,74,75]. For instance, in the Ploutz and Snyder’s [70] study, healthy older women performed eccentric training for a duration of 12 weeks with no reported injuries emerging from the experimental stimuli. Hortobágyi et al. [71] performed a 7 day eccentric overload study including older women, and they reported no injury or dropout as a result of the study protocol. Reeves et al. [58] examined a 14 week adaptation to eccentric training in old men and women and did not report injury emerging from the study protocol. Vincent et al. [21] performed 16 weeks of eccentric training in older men and women with knee osteoarthritis. The authors reported some dropouts but none related to injury emerging from the eccentric training protocol. Gluchowski et al. [63] realized an 8 week study including eccentric training in old men and women. The authors reported five dropouts, including one report of knee soreness (reportedly unrelated to the study protocol). Clark and Patten [74] examined 5 weeks of eccentric training in older adults after stroke and reported one dropout not from the eccentric training group but from the concentric one (unrelated medical conditions). Accordingly, the application of eccentric exercise in older adults seems safe, with no exact injuries related to eccentric training itself. A more detailed review regarding the safety of eccentric training in rehabilitation was performed by La Stayo et al. [76], who, after reviewing several studies, focused on the safety of eccentric training in older adults without limitations and older adults who survived cancer. They did not find any occurrence of injuries in these groups. However, closer attention should be paid to the execution of these eccentric exercises, such as positioning, form, or technique of the exercise, which can place further stress on the joints (ankles, knees, and hips). This may be even more important for older individuals with various diagnoses such as osteoarthritis, osteoporosis, or osteopenia. The presence of a certified and experienced person during any form of eccentric training (as well as other forms) is very important from a safety standpoint; in many (if not all) centers for older individuals, it is already a requirement.

## 2. Conclusions

Our review showed that daily living activities in older adults are highly demanding, even reaching very close to maximal capabilities of older individuals [36]. Several research studies showed that, even during these so-called “simple tasks”, the forces that act on individuals highly exceed their own body weight. These forces are even higher during more demanding tasks such as running or sprinting in the active older population. The presented summaries of eccentric studies in older individuals showed that even older adults can perform eccentric training with intensities ranging from low to high and with low to no prevalence of eccentric-related injuries, highlighting a positive impact on daily living activities. The ability of older adults to handle even higher intensities during eccentric training suggests that, if applied correctly, the resulting effect can counteract the age-related decline in physical functioning and enable them to more easily accomplish a broader range of daily living activities. This may prolong the self-sufficiency of the elderly and improve their quality of life.

## 3. Bullet Points

- Sarcopenia is one of the factors influencing the execution of daily living activities.
- Daily living activities put high demands on older individuals.
- The most demanding DLAs are stair climbing and stair descent.
- The highest demands occur during eccentric phases of movement such as stair descent.
- Evidence shows that eccentric training performed using various modalities is effective to counteract the age-related decline in physical functioning.
- The most frequently used intensity during eccentric training is ≥50% of the maximal eccentric strength; however, it was shown that positive changes occur with intensities ranging from low to high.
- The most optimal frequency of eccentric trainings is 2–3 sessions per week.
- The prevalence of eccentric training-related injuries seems to be low to none.

## Figures and Tables

**Table 1:** Represents summary of studies focused on demands of daily living activities.

Study	Activity	Population	Tests	Methods	Outcomes	Results
Studies related to stair ascent and descent activity						
McFyden et al. [23]	Stair ascent and descent	- Case study—3 males	- 5-step stair ascent and descent	- Kinematic and kinetic assessment - Ground reaction forces	- Ankle, knee and hip moments (Nm.kg^−1^) during ascending and descending	- Data presented in graphs
Kowalk et al. [24]	Stair ascent and descent	- Mixed group of men and women ranging from young to middle age (22 to 40 years old)	- 3 sets of 3 repetitions stair ascending and descending starting with left and right foot	- kinematic and kinetic measurement (force plates)	- Knee joint moments normalized to body weight and leg length expressed as % difference	- Real data converted to Nm only in graph
Leitner et al. [25]	Stair ascent and descent	- Young males, ~28 years	- 3 stair ascents and descents	- Vertical ground reaction force (VGFR)	- Newton (N)	Stair ascent right leg: - Fmax1: 662.9 N - Fmax2: 743.1 N Stair ascent left leg: - Fmax1: 665.1 N - Fmax2: 735.9 N Stair descent right leg: - Fmax1: 1011.8.9 N - Fmax2: 639.9 N Stair descent left leg: - Fmax1: 953.9 N - Fmax2: 636.8 N
Christina-Cavan [26]	Stair descent	- 12 young males and females: 24 ± 3 years - 12 older males and females: 73.3 ± 1.9 years	- 7-step stair descent test with different illumination	- Force plates, photocells - 5 trials	- Ground reaction forces (GRF)	- Young: 1.40–1.48 BW (2 highest force peaks) - Elderly: 1.43–1.50 BW (2 highest force peaks)
Larsen et al. [27]	Stair ascent and descent	- 19 elderly women (72.3 ± 6.6 years) - 11 young women (25 ± 8 2 years)	- 9-step staircase - ascending and descending with self-selected, standardized or maximal pace/velocity	- Force plate	- Ground reaction forces normalized to % BW	- Exact data regarding GRF load presented in the graphs. - According to data in the graphs, values regarding GRF for ascending were about 100% BW in older women and 110% BW in young women with standardized velocity - For descending phase values were about 130% BW in older women and 130% BW in young women with standardized velocity
Samuel et al. [28]	Stair ascent and descent	- 84 older adults (73.2 ± 7.3 years) - 41 males - 43 females	- Maximal strength assessment of knee flexion and extension - Stair ascent - Stair descent	- Torque dynamometer - 3D motion analysis system - Force platforms	- Peak joint moments (Nm/kg) - Functional demands of joints expressed as % of maximal force production	- Stair ascent: knee extensors—102.9%, knee flexors 42.2%, hip extensors—88.9%, hip flexors 42.7% - Stair descent: knee extensors—120.4%, knee flexors 73.3%, hip extensors—50.6%, hip flexors 43.3%
Studies related to walking and running activity						
Kulmala et al. [29]	Walking, running, sprinting	- 13 old individuals (~67 years) - 13 young men (~27 years) - with years of training experience in track and field	- Knee and ankle forces during walking, running, sprinting, hopping reference test	- 3D motion analysis - Force platforms	- Peak vertical ground reaction forces (also many other metrics) - Normalized to bodyweight	- Walking old: 1.27 ± 0.09 BW - Walking young: 1.23 ± 0.06 BW - Running old: 2.85 ± 0.30 BW - Running young: 3.11 ± 0.31 BW - Sprinting old: 2.93 ± 0.32 BW - Sprinting young: 3.76 ± 0.51 BW - Hopping test old: 2.93 ± 0.54 BW - Hopping test young: 3.76 ± 0.51 BW
Messier et al. [30]	Walking	- 142 sedentary overweight and obese older adults with osteoarthritis with age 68.5 ± 0.52 years	- Relationship between weight loss and knee joint kinetics - Gait analysis	- 3D gait analysis - videography - Force platforms	- Peak knee joint forces - Peak internal knee joint moments	- Unadjusted baseline peak knee forces: anterior-posterior shear force 475.9 ± 16.2N, compressive force 2892.2 ± 73.0 N, resultant force 2926.2 ± 73.5 N
Watt et al. [31]	Traditional and treadmill walking	- 18 elderly subjects (65 to 81 years)	- 15 m overground walking (1.27 m/s) - Treadmill walking (1.25 m/s)	- Treadmill force plates (in-built) - Grounded force platforms - 3D motion analysis system	- Treadmill ground reaction forces - Ground reaction forces	- Vertical GRF ranged from 100.23% BW to 111.25% BW (Included 2 peak/phases)
Buddhadev et al. [32]	Walking	- 18 older adults (71.8 ± 5.8 years) - 16 young people (25.3 ± 4.3 years)	- 10m walking - 3 fixed speeds (1.1, 1.3, and 1.5 m·s^−1^)	- Gait mat - Force platforms	- Net extensors joint moments - normalized to bodyweight and leg length (%BWxLL)	- Data in graph - Ankle joint moment: ~8x (%BWxLL) - Knee joint moment: ~3–4x (%BWxLL) - Hip joint moment: ~3–4x (%BWxLL)
Studies with combined activities						
Luepongsak et al. [33]	Standing, walking, chair raise, stair descent, bending	- 132 elderly people - 74.9 ± 6.5 years	- 7 s standing task - Chair raise - 10 m walking - Bending down to reach an object - 4-step stair descent - 2 trials per task	- 3D full body motion analysis with optoelectrical camera - Force platforms	- GRF (% BW)	- Highest forces found in knees and hips during stair descent (knee: 123.58% BW and hip: 108.74% BW) - walking (knee: 101.03% BW and hip: 88.75% BW)
Stacoff et al. [34]	Walking, stair ascent and descent	- Twenty subjects of three age groups (young 33.7 years; middle 63.6 years; old 76.5 years	- 25 m of level walking - Flat, standard, and steep staircase (6 to 9 steps)	- Kinematic and kinetic data - Force platforms	- Vertical GRF normalized to BW	- Average GRF during stair descent increased up to 1.62–1.67 BW in young and 1.51–1.53 BW in elderly (listed values in the range because of left and right leg)
Taylor et al. [35]	Gait and stair climbing	- 4 total hip arthroplasty patients (~61 years)	- Gait analysis of walking and stair climbing	- Force platforms - optical system using cameras - The in vivo hip contact force was measured in the femoral coordinate system	- GRF	- The average peak tibio-femoral contact forces during walking were 3.33, 3.23, 3.02, and 2.97 BW across the four patients - The maximum averaged forces during stair climbing were 5.88, 5.23, 5.09, and 5.33 BW for the four patients
Hortobágyi et al. [36]	Stair ascent and descent, chair stand, isometric leg-press	- 13 young adults (22 ± 2 years) - 14 old adults (74 ± 3 years)	- 4-step stair ascent and descent - Sit-to-stand task - Isometric maximal leg strength	- Force platform - Video analysis - EMG analysis - Leg-press machine	- Knee joint moments - Knee joint kinematics	Ascent: - Peak extension moments were 1.55 ± 0.24 and 1.00 ± 0.24 N·m/kg in young and old, respectively. Descent: - Peak extension moments were 0.90 ± 0.24 and 0.64 ± 0.24 N·m/kg in young and old, respectively. -Old individuals closer to their maximal capacity compared to the young ones
Kutzner et al. [37]	Stair ascent, walking, one- and two-legged stance, standing up and sitting down, knee bending	- 4 males and 1 female (60 to 71 years) - in vivo	- Two-legged stance - Sitting down - Standing up - Knee bend - One legged stance - Level walking - Ascending stairs - Descending stairs	- The instrumented knee implant measured the 3 contact forces and 3 contact moments, acting on the tibial component	- Contact forces (F_z_, F_x_, F_y_) - Contact moments (M_z_, M_x_, M_y_) - Calculated resultant forces	- 2-legged stance:107% BW - Sitting down: 225% BW - Standing up, knee bend, 1-legged stance, level walking: 246–261% BW - Stair ascending 316% BW - Stair descending 346% BW - Maximum measured force was 400% BW during stair descending
Hamel et al. [38]	Stair ascent and descent, walking	- 12 young women (24.3 ± 2.5) - 10 older women (73.5 ± 2.6)	- 7-step stair ascent - 7-step stair descent - 10 m Overground walking - Fixed speeds	- Force plates - Photocells	- Ground reaction forces (normalized to BW) - Vertical forces	- Ascent: young 1.16 ± 0.08 BW, elderly 1.04 ± 0.07 BW - Descent: young 1.42 ± 0.12 BW, elderly 1.50 ± 0.25 BW
Heinlein et al. [39]	Walking, stair climbing	- Clinical study on 2 subjects with telemetric tibial tray - in vivo	- Level walking - Stair ascent and descent	- Telemetric tibial tray	- Contact forces (F*_z_*, F*_x_*, F*_y_*) and moments forces (M*_z_*, M*_x_*, M*_y_*) - Calculated resultant forces	- level walking: −208/−276% BW - stair ascent: −166/−223% BW - stair descent: −352/−327% BW (without support 6 to 10 month post operation)
Samuel et al. [40]	Chair raise, stair ascent and descent, gait	- 84 older adults (73.2 ± 7.3 years)	- Muscular strength: knee and hip extensors and flexors - Functional demands: gait, stair ascent and descent, chair raise, sitting down	- Torque dynamometer	- Functional demands calculated as % of maximal force production achieved in maximal strength test	Only maximal values presented: - Knee extensor demand of gait: −101.1% - Hip extensor demand of gait: −127% - Knee extensor demand of stair ascent: −102.9% - Hip extensor demand of stair descent: −120.4%

*Note:* 3D—three-dimensional, Nm—newton-meters, Nm·kg^−1^—newton meter to kilogram-force, %—percentage, BW—bodyweight, GRF—ground reaction force, s—second, m—meter, EMG—electromyography, F*_z_* F*_x_*, and F*_y_* represent superior-inferior, medial-lateral, anterior-posterior GRF and M*_z_*, M*_x_*, M*_y_* moments, m/s—meters per second, %BWxLL—percentage normalization to bodyweight and leg length.

## Data Availability

All the data presented in this review are related to the studies included in the text and table.
